# Influence of Personal Experiences of Medical Students on Their Assessment of Delivering Bad News

**DOI:** 10.3390/ijerph191912040

**Published:** 2022-09-23

**Authors:** Agata Kotłowska, Julia Przeniosło, Krzysztof Sobczak, Jan Plenikowski, Marcin Trzciński, Oliwia Lenkiewicz, Julia Lenkiewicz

**Affiliations:** 1 Faculty of Medicine, Student Scientific Circle of Medical Communication at the Medical University of Gdansk, 80-210 Gdansk, Poland; 2 Department of Sociology of Medicine and Social Pathology, Medical University of Gdansk, 80-210 Gdansk, Poland

**Keywords:** delivering bad news, diagnosis, truth disclosure, doctor–patient relationship, medical communication

## Abstract

Background: We aimed to identify which attitudes and emotions accompany latter-year medical students as they experience situations where bad news is communicated. Methods: A cross-sectional study was conducted using the computer-assisted web interview (CAWI) methodology in a group of 321 fifth- and sixth-year medical students from 14 medical universities in Poland. Correlations were analyzed using Pearson’s χ^2^ test. For the categorical variables, subject profiles were analyzed using K-means clustering. Results: Students’ self-assessments of their competence in delivering bad news (DBN) differed depending on the type of experience they had with it. More than half of the students had observed a situation of DBN (63.6%) and as many as 26.5% of the participants had received bad news themselves. These two groups were less likely to declare a lack of DBN-related skills (43.4% and 33.4%, respectively) than others. In this study, 9% of the students had personally delivered bad news. Only 13.4% of these students rated their DBN skills as insufficient. They were also the least likely to express concern regarding high levels of stress (29.6%) and anxiety (48%). Conclusions: The ability to personally deliver bad medical news to a patient was the most effective form of gaining experience in DBN. Being a bearer of bad news may help students develop their own strategies for coping with difficult emotions and develop their professional competences, leading to improved medical care and patient comfort.

## 1. Introduction

Bad medical news can be defined as any news which directly or indirectly translates into a negative change in the patient’s life and permanently degrades their quality of life [1]. Numerous studies have shown that both the receipt and the delivery of unfavorable messages are linked to high levels of stress and psychophysical burden [2,3,4]. A number of strategies have been implemented to support doctors by reducing the impact of these experiences. Evaluations of training courses aimed at developing competence in delivering bad news (DBN) suggest increased efficacy of communication behaviors [5], better empathy [6], reduced stress [7], and a stronger sense of one’s own professionalism [8].

The way in which bad news is delivered to patients is known to have a significant impact on their behavior [2]. Ineffective DBN strategies negatively affect the perception of a disease and translate into therapeutic outcomes [1]. Patient-centered care requires an approach in which the needs of patients and their families are taken into account [9]. This is one of the reasons behind the importance of effective communication strategies. Training and practice in this regard are required as early as in undergraduate medical education [10].

Mandatory teaching of DBN-related skills is necessary, although it is also a difficult educational experience for students. Observations of students’ psychophysical parameters during simulated DBN situations reveal acute reactions associated with high stress levels [11,12]. Cognitive analysis confirms the nature of these complex experiences; however, with this form of training, students can be faced with their own emotions and develop optimum coping strategies [13].

Numerous reports are available that reveal the emotional concerns of students regarding DBN [14,15,16]. Studies show that Polish medical students as well as medical students from other countries experience high anxiety levels and express concerns connected to delivering bad news [17]. However, there is a striking shortage of studies analyzing the impact of real DBN experience on students’ attitudes.

When designing our study, we began with the following research question: What attitudes and emotions might accompany students as they experience situations where bad news is communicated? The objective of our study was to compare how being the recipient, observer, or deliverer of bad medical news affected the attitudes and emotions of students. We wanted to learn how these experiences affected the self-perception of being prepared for the delivery of bad news among latter-year medical students. This study might contribute to improvements in the training of medical students in delivering bad news to patients.

## 2. Materials and Methods

### 2.1. Study Design

A cross-sectional study was conducted by the quantitative method using the computer-assisted web interview (CAWI) methodology. The participants completed a questionnaire, made available on a website dedicated to research (www.ebadania.pl, accessed on 1 July 2021). The data were collected using a proprietary research tool that was approved by the Independent Bioethics Committee of the Medical University of Gdańsk (No. NKBBN/287/2021). The digitized questionnaire was published on a dedicated, professional website. The website was designed to block IP addresses so that no participant would be able to answer the questionnaire more than once. Participation in the study was selective and the collected data were anonymous.

Using the survey questionnaire, we asked medical students about their experience and preparedness for transmitting unfavorable medical information. The questionnaire consisted of thirty-one questions. For presented results we used fourteen close-ended questions. In this report, we present the results pertaining to the respondents’ experiences, emotions, and concerns related to DBN situations. Those were the dependent variables. The independent variables consisted of eight questions regarding gender, age, year of study, medical school, patient contact experience (other than that obtained as part of clinical training) and personal experience in receiving bad medical news.

### 2.2. Setting

The data were collected between 15 April and 1 July 2021. The participation was anonymous. Voluntary consent for participation in the study was confirmed by each participant. No sensitive data were collected as part of the study. The study manual contained information on the study’s purpose and form, as well as the subjects’ right to withdraw at any stage of the study. The study population was targeted by means of the administrative structures of medical universities in Poland, bulletins, research circles, student councils, and social media with a nationwide reach.

### 2.3. Participants

A total of 321 contributions from respondents were collected and each one was included in the analysis. The selection of the subjects was random and the inclusion criterion was being a fifth- or sixth-year student at a medical faculty. Ultimately, students from 14 Polish medical schools took part in the study.

According to official government statistics, in the 2020–2021 academic year 21 universities in Poland offered an undergraduate program in medicine to approximately 34,000 students [18]. Unfortunately, the number of students in the fifth and sixth year was not provided. Therefore, we could not determine the saturation level for the study group. The study group in this research was not a quota sample.

### 2.4. Variables

Independent study variables included the respondents’ gender, age, year of study, medical school, personal experience in receiving bad medical news, preferences regarding future specialization, and patient contact experience other than that obtained as part of their clinical training.

The variables analyzed in this study were related to nine closed-ended questions regarding the students’ experience and concerns related to DBN. A Likert scale was used in seven of these questions. In our analysis of the responses, the categories “Definitely not” and “Rather not” were pooled into a single negative category. The same scheme was used for the responses “Yes, absolutely” and “Yes, somewhat”, which were pooled into a single positive category.

### 2.5. Statistical Methods

Statistical analysis was carried out using the software Statistica v. 13.3 (StatSoft, Tulsa, OK, USA). We used Pearson’s χ^2^ test to analyze the relationships between the nominal variables obtained in the questions with the Likert scale, with the significance level set at *p* < 0.05. For the cluster analysis, we used the K-means analysis. Thanks to the PCA (Principal Component Analysis), it is possible to measure simple effects. The proposed method has yet another advantage. It makes predictions possible. This means that if in the study sample, a combination of immersive experiences has not occurred, we can then analyze individual answers and assess the risk of not only an individual occurrence, but also any combination of co-occurrences [19,20,21].

## 3. Results

The study group consisted of 321 fifth- and sixth-year students of 14 medical universities in Poland (Table 1). The study group featured an over-representation of women, as the percentage of female subjects was 12.7% higher than the percentage of all women studying at Polish faculties of medicine [18]. Students of the Medical University of Gdańsk were also significantly overrepresented in the study sample.

In order to analyze the students’ self-evaluation of their preparedness for delivering bad medical news, we asked them about the types of their direct experience with these situations. The responses were analyzed for the form of experience:

- being a patient and personally receiving bad news, were the group we referred to as receivers;
- participating in clinical training and witnessing physicians delivering bad news to patients, were referred to as observers; and
- personally providing patients with unfavorable information, were referred to as deliverers.

Exactly 149 students (46.4%, n = 321) admitted that they had experience in one of the above categories. Experience in two categories was reported by 71 students (22.1%, n = 71), and nine students (2.8%) declared personal experience in all three categories.

### 3.1. Bad News Receivers

As many as 85 students (26.5%) declared that they had been in a situation where they had personally received news about an unfavorable diagnosis from a physician. We discovered that this experience influenced their assessment of their own competences in the field of DBN. Students in this group were less likely to declare a lack of DBN-related skills (33.4%, n = 18) as compared to other students (54.6%, n = 84; χ^2^ = 7.198; df = 1; *p* = 0.007). We found no statistically significant correlations when comparing the type of experience with other variables (Table 2).

### 3.2. DBN Observers

As many as 204 (63.6%) students within the study group declared having observed a physician delivering bad news to a patient as part of their clinical training. The experience had an educational value as well as a positive impact on the students’ self-assessed competences. Students who had experienced the opportunity to observe the delivery of bad news were less likely to declare an inability to deliver unfavorable medical information (43.4%; n = 56) than those who had not had such an experience (58.2%; n = 46; χ^2^ = 4.304; df = 1; *p* = 0.038). As with our findings in the subgroup of receivers, no significant correlations between the remaining variables were identified among the observers.

### 3.3. Bad News Deliverers

A total of 29 (9%) students had found themselves in a situation where they personally delivered bad medical news to a patient. This type of experience proved to have the greatest educational impact (Table 3). The vast majority of students within this sub-group had experience beyond the minimum required to pass clinical training (eight students had worked as physicians’ assistants, 10 had additional experience from working in student research circles, two students had been involved in volunteer medical work, and five students declared having relevant experience from other sources). Only four of the deliverers declared that their patient contact experience to date had been limited to mandatory clinical training.

The students who had personally delivered bad medical news were less likely to declare themselves unable to deliver unfavorable information (13.6%, n = 3) than others (53.2%, n = 99) (χ^2^ = 12.338; df = 1; *p* < 0.001). They were also less frequently (31%, n = 9) afraid of the patient’s emotional reaction to an unfavorable diagnosis (70%, n = 194; χ^2^ = 17.883; df = 1; *p* < 0.001). Moreover, stress at the thought of having to deliver bad medical news was less frequent in this group (60.4%, n = 160; χ^2^ = 9.482; df = 1; *p* = 0.002), as were concerns regarding potentially delivering bad news without the appropriate tactfulness and prudence (48%, n = 12) (71.5%; n = 181; χ^2^ = 5.940; df = 1; *p* = 0.014). One can therefore conclude that the experience of providing unfavorable messages as part of clinical training effectively reduces negative emotional experiences and, to some extent, enhances the sense of professionalism.

### 3.4. Self-Assessment of Skills and Concern Profiles

K-means clustering was used to group the subjects’ experience profiles into three clusters (Table 4). The scale reliability analysis for all questions was α = 0.700.

The first profile (1) encompassed more than one half of all the subjects (59.8%). The students declared that they were unable to provide unfavorable medical news. At the same time, they believed that showing empathy when DBN would be challenging for them. They also reported being afraid of patients’ emotional response. The students were not sure whether they would meet the expectations of patients regarding tactfulness and prudence. The students in this profile experienced stress at the mere thought of having to carry out such conversations with their patients.

The second profile (2) encompassed about one quarter of the subjects. The students in this group did not agree with the statement that they were unable to deliver unfavorable medical information. They were concerned about the emotional responses of patients, yet declared that they themselves were not afraid of the stressful experiences associated with patient contact as part of the DBN process.

The last profile (3) encompassed those students who remained unsure of their DBN competencies, allowing them to pass on the bad news in a tactful and prudent manner.

## 4. Discussion

There are 21 universities in Poland that offer an undergraduate course in medicine. Undergraduate Medicine in this country is a six-year-long degree (12-semester). The classes during the first three years of the course are mainly theoretical, while practical training only begins in the fourth year; this is when students get the chance to acquire communication skills during clinical subjects. For this reason, we collected data solely from fifth- and sixth-year medical students in our research. DBN courses in most cases are not compulsory and are not part of the curriculum.

The fifth- and sixth-year undergraduate medical students assessed their DBN-related skills as insufficient. More than 70% of the respondents had participated in a DBN situation. Depending on the type of experience, the subjects presented with varying awareness of the emotions they might encounter in these situations. Most respondents (59.9%) associated DBN with a risk of stress and fear over the patient’s reaction.

### 4.1. Receivers and Observers

As many as 85 (26.5%) students had personally had a physician deliver the news of an unfavorable diagnosis to them (we refer to these students as recipients). A minority of these students (33.4%) declared a lack of skills in delivering bad medical news.

More than 60% of the respondents declared having observed bad medical news being delivered (we refer to these students as observers). The students in this group were also less likely to declare being incompetent to deliver bad medical news (43.4%).

Both observers and receivers noted that the experience of observing a physician giving a patient an unfavorable diagnosis had contributed to their positive self-assessment of their own communication skills. Other studies support the view that students’ participation in the communication course as observers and as active participants leads to higher confidence [22]. Thus, one may conclude that these types of experiences in DBN are educationally valuable. However, being convinced that one has the skills is not enough to deliver bad news properly. Undergoing DBN training can significantly increase one’s self-confidence [23], although it does not necessarily mean that the required competencies have been mastered. Students may declare an ability to communicate with patients in an appropriate manner, though there may be no correlation between their skills and self-assessment [24]. The self-assessment of communication skills is largely determined by clinical experience. In a study by M. H. Brouwers, students of the latter years, having had more experience contacting patients, were more aware of their incompetence than fourth-year students [25]. This type of awareness may be an important starting point for the actual development of communication skills. Neither being an observer of DBN nor having the personal experience of receiving bad news has any impact on the development of communication skills. As K. Woolf et al. demonstrated, a patient’s perspective may be useful when students are to play the roles of physicians. Although the experience of one’s own illness significantly increases the level of anxiety of medical students, it positively affects the level of empathy toward patients in physicians [26]. In our study, we noted a positive correlation between being the recipient of bad news and a lower likelihood of declaring a lack of DBN-related skills. We believe that the situation of being a patient can increase a student’s self-assessment of their competences in the area of DBN. There is an educational potential that we believe can be used to develop these dispositions. Studies based on the premises of narrative medicine revealed that the use of the “reflective writing” method contributes to higher levels of empathy. Medical students taking part in this kind of training reported their personal experience of severe illness or a severe illness of a close relative, reflecting on the emotions they experienced in those situations [27,28]. Intrapersonal communication is an important and useful competence in clinical practice [29]. It appears that students who have been the recipients of bad news, and thus have experienced the patient’s perspective, can have a greater understanding of patients through their own experiences. Their emotional experience, if properly worked upon, may become an important foundation for the development of decentration and empathy.

The experience of being the recipient or observer of DBN, although it has no effect on the development of practical skills, is important due to its potential in enhancing empathy and improving one’s sense of one’s competences. We think that it is worth considering “immersing” students in the experience of being an observer, even during the first years of undergraduate medical education. This type of experience may prove valuable, not only for cognitive, but also for emotional development.

### 4.2. Deliverers

In our research, we observed that students who had experience delivering bad medical news constituted a completely different subgroup of subjects; one characterized by the awareness of their own competences. Exactly 29 subjects (9%) declared that they had delivered bad medical news to patients. Within this group, only three individuals rated their DBN skills as insufficient. The experience of being the bearer of bad news was negatively correlated with anxiety, stress, and fear of the patients’ emotional reactions. From the perspective of our study, DBN can be described as the most productive type of experience. Students in this subgroup demonstrated a higher sense of their own competence in DBN. They reported lower levels of anxiety and increased attentiveness to the way in which the diagnosis was delivered. From the standpoint of education, we believe that the introduction of mandatory DBN experience is worth considering. The experience, supervised by an experienced physician, should be offered to latter-year students as a prerequisite for the completion of the degree program. Since DBN requires an encounter with a real patient, which might be stressful and may involve a high emotional burden for the student delivering the bad news, we suggest that such an experience should involve appropriate preparation. A similar situation, such as role-playing scenes with a standardized patient (SP), should be considered. Conversations with an actor playing the role of the patient may provide the students with an opportunity to try out different notification techniques and to verify dedicated DBN protocols [30]. Analyzing a recording of the conversation with an SP allows the student to observe the situation. Moreover, any mistakes that have been made do not affect the real patient, ensuring a comfortable learning experience [31]. Evaluations of educational interventions using the SP method show an increase in the empathy levels and students’ communication skills [32,33].

However, the limitations of this method should also be taken into account. Some studies have demonstrated the disadvantages of this technique by pointing to the relationship being reversed in terms of power and knowledge [34]. When delivering bad news, the physician has greater knowledge and the right of insight into the patient’s intimate sphere. In a simulated situation, it is the SP (actor) rather than the bad news deliverer (student) who defines the framework for the discussion. The actor knows the scenario (including the diagnosis), which places them in a superior position relative to the student. This is why delivering bad news to a real patient ensures authenticity that cannot be formed during contact with an SP. While delivering bad news to a real patient, the position of the student is closest to that of the physician and facilitates actual “immersion” in the emotions of a bad news deliverer.

Participating in DBN may be stimulating for both observers and deliverers. By watching a physician deliver bad medical news, students are capable of experiencing the emotions of actual patients, along with their own. In our study, the observers self-assessed their own competences more positively. This component of attitude is important for finding the courage to engage in the role of deliverer. However, the experience of delivering bad news to a patient may not be sufficient to develop complementary communication skills. Joanne M. Shaw demonstrated that having more professional experience does not reduce the stress associated with DBN. Developing appropriate techniques for coping with difficult emotions appears to be more important than experience in this regard [35]. DBN is a type of immersion, allowing the student to develop their own strategies for dealing with emotions. Students who had delivered bad news declared lower levels of stress and anxiety than others who had no such experience.

Our study revealed a significant educational value of incidental education in relation to DBN. Personal experience of students can only become an important element in the development of professional competence if it is taken into account in the formal education process. We are also aware of the objective limitations of our study. When interpreting the results, one should remember that the questions addressed to students were prospective in nature. There was also a significant overrepresentation of students from the Medical University of Gdańsk. Density distribution analysis revealed a relatively small number of students with DBN experience as compared to other studies. In those studies, DBN experience was reported for up to 50% of the students participating in similar experiments [31]. Such differences may result from different standards of education in medical schools in individual countries, which should be taken into account when interpreting the results of this study. Additionally, it is important to consider the varying curricula between different medical universities in Poland.

## 5. Conclusions

We discovered that being the recipient, observer, or deliverer of bad news has a positive effect on students’ sense of preparedness for DBN. The results show that only the experience of being a bad news deliverer lowered the medical students’ anxiety and stress issues.

The opportunity to deliver bad news during clinical training can effectively contribute to the development of communication skills and strategies for coping with difficult emotional experiences in a safe environment. Observing bad news being delivered by a physician also affects a students’ self-assessed sense of preparedness for DBN. Students who had received bad news themselves declared a lack of DBN-related skills less frequently than other students.

Whatever the type of experience in DBN, it is of educational value. Medical students with no personal experience with DBN notification may be at risk of a greater sense of anxiety, professional uncertainty, and unpreparedness for DBN. In our view, eliminating this kind of educational gap can help improve the vocational training of students and thus improve the quality of medical care and patient comfort.

## Figures and Tables

**Table 1:** Characteristics of respondents.

Categories		N (%)
**Gender**	Female	232 (72.3%)
	Male	87 (27.1%)
**Age**	23–24 years	152 (47.4%)
	25 years or older	169 (52.6%)
**Year of study**	Fifth year	165 (51.4%)
	Sixth year	156 (48.6%)
**Experience in patient contact ^1^**	Physician’s assistant	44 (13.7%)
	Volunteer	26 (8.1%)
	Working in scientific circles	66 (20.6%)
	Other than the above	56 (17.4%)
	No experience	129 (40.2%)
**Medical school**	Medical University of Gdańsk	112 (34.9%)
	Medical University of Warsaw	38 (11.8%)
	Medical University of Łódź	34 (10.6%)
	Jagiellonian University Medical College	30 (9.3%)
	Wrocław Medical University	23 (7.2%)
	University of Nicolaus Copernicus Medical College	18 (5.6%)
	Pomeranian Medical University in Szczecin	16 (5%)
	Poznań University of Medical Sciences	13 (4%)
	Medical University of Silesia	11 (3.4%)
	Other	26 (8.1%)
**Personal experience with DBN**	Bad news recipients ^2^	85 (26.5%)
	DBN observers ^2^	204 (63.6%)
	Bad news deliverers ^2^	29 (9.03%)
	No experience	92 (28.7%)

^1^ in addition to mandatory clinical training. ^2^ responses in more than one category.

**Table 2:** Responses regarding DBN-related concerns.

Categories	N (%)		
	Yes	No	I Don’t Know
I am unable to provide unfavorable medical news.	102 (31.8%)	106 (33.0%)	113 (35.2%)
I fear the patient’s emotional reaction to an unfavorable diagnosis.	203 (63.2%)	103 (32.1%)	15 (4.7%)
Showing empathy during DBN will be challenging for me.	56 (17.4%)	231 (72.0%)	34 (10.6%)
I do not know if I can deliver bad news with the tactfulness and prudence the patient expects.	193 (60.1%)	85 (26.5%)	43 (13.4%)
I feel stressed at the mere thought of having to face such conversations.	168 (52.4%)	124 (38.6%)	29 (9%)

**Table 3:** Assessment of personal experience in DBN.

Questions and Response Categories	N (%)	
**Has your personal DBN experience been stressful to you?**		
Yes, absolutely	7 (24.1%)	21 (72.4%) ^1^
Yes, somewhat	14 (48.3%)	
It is difficult to say	4 (13.8%)	
Rather not	3 (10.3%)	4 (13.8%) ^2^
Definitely not	1 (3.4%)	
**Do you think you have delivered the news of a patient’s unfavorable prognosis in an appropriate manner?**		
Yes, absolutely	4 (13.8%)	18 (62.1%) ^1^
Yes, somewhat	14 (28.3%)	
It is difficult to say	9 (31%)	
Rather not	2 (6.9%)	2 (6.9%) ^1^
Definitely not	0 (0%)	

^1^ Total for “Yes, absolutely” and “Yes, somewhat”. ^2^ total for “Definitely not” and “Rather not”.

**Table 4:** Independence test for qualitative variables with profile clusters (N = 321).

Categories	df	χ^2^	*p*	G^2^	R^2^	*p* *	Profile Clusters		
							1	2	3
I am unable to provide unfavorable medical news. ^3^	4	190.658	<0.001	196.394	156.543	<0.001	I agree ^1^	I do not know	I do not agree ^2^
I fear the patient’s emotional reaction to an unfavorable diagnosis. ^3^	4	111.625	<0.001	122.558	132.533	<0.001	I agree ^1^	I do not agree ^2^	I agree ^1^
Showing empathy when DBN will be challenging for me.	4	13.436	<0.001	14.472	11.452	0.005	I do not agree ^2^	I do not agree ^2^	I do not agree ^2^
I do not know if I can deliver bad news with the tactfulness and prudence the patient expects. ^3^	4	250.339	<0.001	225.324	212.345	<0.001	I agree ^1^	I do not know	I do not agree ^2^
I feel stressed at the mere thought of having to face such conversations. ^3^	4	140.910	<0.001	153.157	134.434	<0.001	I agree ^1^	I do not agree ^2^	I do not agree ^2^
N							192	47	82
(%)							59.8%	14.6%	25.5%

df—“degrees of freedom”; χ^2^—“chi-squared distribution; *p*—“*p*-value”; G^2^—“likelihood-ratio test”; R^2^—“coefficient of determination”. * the result is reliable at the level of α = 0.05. ^1^ total for “Yes, absolutely” and “Yes, somewhat”. ^2^ total for “Definitely not” and “Rather not”. ^3^ reliability level α = 0.764.

## Data Availability

The datasets used and/or analyzed during the current study are available from the corresponding author on reasonable request.
